# COVID-19 Vaccination Coverage and Factors Associated With Vaccine Uptake Among People With HIV

**DOI:** 10.1001/jamanetworkopen.2024.15220

**Published:** 2024-06-06

**Authors:** Rulin C. Hechter, Lei Qian, In-Lu Amy Liu, Lina S. Sy, Denison S. Ryan, Stanley Xu, Joshua T. B. Williams, Nicola P. Klein, Robyn M. Kaiser, Elizabeth G. Liles, Jason M. Glanz, Lisa A. Jackson, Maria E. Sundaram, Eric S. Weintraub, Hung Fu Tseng

**Affiliations:** 1 Kaiser Permanente Southern California, Pasadena, California; 2 Kaiser Permanente Bernard J. Tyson School of Medicine, Pasadena, California; 3 Denver Health, Denver, Colorado; 4 Kaiser Permanente Vaccine Study Center, Oakland, California; 5 HealthPartners, Bloomington, Minnesota; 6 Kaiser Permanente Center for Health Research, Portland, Oregon; 7 Institute for Health Research, Kaiser Permanente, Denver, Colorado; 8 Kaiser Permanente Washington Research Institute, Seattle; 9 Marshfield Clinic Research Institute, Marshfield, Wisconsin; 10 Centers for Disease Control and Prevention, Atlanta, Georgia

## Abstract

**Importance:**

People with HIV (PWH) may be at increased risk for severe outcomes with COVID-19 illness compared with people without HIV. Little is known about COVID-19 vaccination coverage and factors associated with primary series completion among PWH.

**Objectives:**

To evaluate COVID-19 vaccination coverage among PWH and examine sociodemographic, clinical, and community-level factors associated with completion of the primary series and an additional primary dose.

**Design, Setting, and Participants:**

This retrospective cohort study used electronic health record data to assess COVID-19 vaccination information from December 14, 2020, through April 30, 2022, from 8 health care organizations of the Vaccine Safety Datalink project in the US. Participants were adults diagnosed with HIV on or before December 14, 2020, enrolled in a participating site.

**Main Outcomes and Measures:**

The percentage of PWH with at least 1 dose of COVID-19 vaccine and PWH who completed the COVID-19 vaccine primary series by December 31, 2021, and an additional primary dose by April 30, 2022. Rate ratios (RR) and 95% CIs were estimated using Poisson regression models for factors associated with completing the COVID-19 vaccine primary series and receiving an additional primary dose.

**Results:**

Among 22 058 adult PWH (mean [SD] age, 52.1 [13.3] years; 88.8% male), 90.5% completed the primary series by December 31, 2021. Among 18 374 eligible PWH who completed the primary series by August 12, 2021, 15 982 (87.0%) received an additional primary dose, and 4318 (23.5%) received a booster dose by April 30, 2022. Receipt of influenza vaccines in the last 2 years was associated with completion of the primary series (RR, 1.17; 95% CI, 1.15-1.20) and an additional primary dose (RR, 1.61; 95% CI, 1.54-1.69). PWH with uncontrolled viremia (HIV viral load ≥200 copies/mL) (eg, RR, 0.90 [95% CI, 0.85-0.95] for viral load 200-10 000 copies/mL vs undetected or <200 copies/mL for completing the primary series) and Medicaid insurance (eg, RR, 0.89 [95% CI, 0.87-0.90] for completing the primary series) were less likely to be fully vaccinated. By contrast, greater outpatient utilization (eg, RR, 1.07 [95% CI, 1.05-1.09] for ≥7 vs 0 visits for primary series completion) and residence in counties with higher COVID-19 vaccine coverage (eg, RR, 1.06 [95% CI, 1.03-1.08] for fourth vs first quartiles for primary series completion) were associated with primary series and additional dose completion (RRs ranging from 1.01 to 1.21).

**Conclusions and Relevance:**

Findings from this cohort study suggest that, while COVID-19 vaccination coverage was high among PWH, outreach efforts should focus on those who did not complete vaccine series and those who have uncontrolled viremia.

## Key Points

**Question:**

What is the COVID-19 vaccination coverage among people with HIV (PWH), and what factors are associated with completion of the primary vaccination series and an additional primary dose?

**Findings:**

In this cohort study of 22 058 PWH, coverage with the COVID-19 vaccine primary series and an additional primary dose was 90.5%. Persons with uncontrolled viremia (viral load ≥200 copies/mL) were less likely to be fully vaccinated, while influenza vaccination in the last 2 years, greater outpatient utilization, and residence in counties with higher COVID-19 vaccine coverage were associated with vaccination completion.

**Meaning:**

Findings suggest that vaccination outreach efforts should focus on PWH who did not complete COVID-19 vaccine series, particularly those with uncontrolled viremia.

## Teaser

This cohort study assesses COVID-19 vaccination coverage among people with HIV and investigates the sociodemographic, clinical, and community-level factors associated with completion of the vaccine primary series and an additional primary dose.

## Introduction

People with HIV (PWH) are at increased risk for severe COVID-19 disease compared with people without HIV, including increased COVID-19 mortality, as reported in the US, the United Kingdom, and South Africa.^1,2,3,4,5,6^ In multicenter cohort studies, immunodeficiency (ie, lower CD4^+^ T cell counts) appears to be associated with severe COVID-19 disease despite virologic suppression.^3,5^ In addition, many PWH have comorbidities associated with greater risk for a more severe course of COVID-19 disease,^7^ and both COVID-19 and HIV disproportionately affect communities of color.^8^

Regardless of CD4 T cell count or viral load, COVID-19 vaccination is recommended for all PWH.^9^ The COVID-19 vaccination program in the US launched on December 14, 2020, with a recommendation for 2 doses of an mRNA COVID-19 vaccine (ie, the primary series). In February 2021, an adenoviral vector vaccine, Ad26.COV2.S (Janssen COVID-19 vaccine), was recommended as a 1-dose primary series.^10,11^ Later, on August 12, 2021, the US Food and Drug Administration (FDA) amended emergency use authorizations for BNT162b2 (Pfizer-BioNTech) and mRNA-1273 (Moderna) COVID-19 vaccines to authorize administration of an additional primary dose at least 28 days after the primary series in immunocompromised individuals. This was due to emerging data demonstrating that 2 doses of mRNA COVID-19 vaccines were less effective in some immunocompromised people.^12^ In addition, immunocompromised persons accounted for a high proportion (≥40%) of COVID-19 infections among fully vaccinated hospitalized persons in the US and Israel.^13,14^ According to the initial recommendation of the Centers for Disease Control and Prevention (CDC) in November 2021 and their updated recommendation in February 2022 for immunocompromised adults,^15,16^ those who received the primary series of 2 mRNA COVID-19 vaccine doses should receive an additional primary dose at least 4 weeks after the second dose, followed by a booster dose at least 3 months after the additional primary dose. Immunocompromised adults who received a single primary dose of Ad26.COV2.S were recommended to receive an additional primary dose at least 4 weeks after the initial dose and a booster dose at least 2 months after the additional primary dose. The timeline of the COVID-19 vaccination recommendations during the study period is illustrated in eFigure 1 in Supplement 1.

The rate of COVID-19 vaccination coverage among PWH is largely unknown, especially among people of color, persons with advanced HIV or comorbidities, and communities with high infection rates and COVID-19–related mortality. Evaluating COVID-19 vaccination among PWH by demographic and clinical characteristics will help identify disparities in vaccine uptake to inform future vaccine implementation strategies during a pandemic and increase understanding of potential factors important in vaccine safety research. In this study, we evaluated COVID-19 vaccination coverage among a diverse cohort of PWH and examined the associations between sociodemographic characteristics, clinical and HIV-specific factors, and community-level COVID-19 infection and COVID-19 vaccination coverage.

## Methods

We conducted a retrospective cohort study of PWH enrolled in the 8 health care organizations in the Vaccine Safety Datalink (VSD), including Kaiser Permanente (Southern California, Northern California, Colorado, Northwest, and Washington regions), Marshfield Clinic, HealthPartners, and Denver Health. The VSD is a collaborative project between the CDC’s Immunization Safety Office and integrated health care organizations across the US that uses electronic health record data from participating sites to monitor and assess the safety of vaccines.^17^ The study protocol was reviewed and approved by the institutional review boards at all sites, with a waiver of informed consent granted on the basis of criteria under 45 CFR 46.114 and 21 CFR 56.114. Reporting followed the Strengthening the Reporting of Observational Studies in Epidemiology (STROBE) reporting guideline.

### Study Population

The study population included adults (≥18 years of age) diagnosed with HIV on or before December 14, 2020 (index date), with COVID-19 vaccination assessed between December 14, 2020, and April 30, 2022. The HIV status and estimated diagnosis date were ascertained through HIV patient registries, a positive HIV Western blot assay result, or an equivalent HIV confirmatory test. All PWH with continuous enrollment from 6 months prior through 4 months after the index date (through April 13, 2021) were followed up until membership disenrollment or December 31, 2021, to assess the uptake of the COVID-19 vaccination primary series. This enrollment criterion allows for adequate time for all individuals to initiate vaccination and complete a primary series during follow-up. A subset of the PWH who completed the primary series by August 12, 2021 (when the additional mRNA COVID-19 vaccine primary dose was authorized) were further followed up for uptake of additional primary dose through April 30, 2022. Individuals who received non–FDA authorized COVID-19 vaccines were excluded.

### Vaccination Status

Receipt of BNT162b2, mRNA-1273, or Ad26.COV2.S was identified using CVX (vaccine administered) codes (ie, 207, 208, 213, 212, and 217). Receipt of NVX-CoV2373 (Novavax) vaccine was not included, as the study observation period ended before NVX-CoV2373 was officially recommended in the US (July 19, 2022). For this study, completion of the primary series was defined as having received 2 valid doses of BNT162b2 or mRNA-1273, a combination of BNT162b2 and mRNA-1273, or 1 dose of Ad26.COV2.S. We considered the second dose valid if it was administered at least 17 days for BNT162b2 or at least 24 days for mRNA-1273 after the first dose of the specific mRNA COVID-19 vaccine and within the CDC-recommended interval.^18^ Persons who only received 1 dose of mRNA COVID-19 vaccine were considered partially vaccinated.

Completion of the additional primary dose of an mRNA COVID-19 vaccine was assessed based on the CDC’s recommended interval and grace period (ie, at least 24 days after completion of the primary series).^16^ Further, uptake of a monovalent booster dose was assessed based on the CDC’s recommended interval for the booster dose for immunocompromised persons (at least 3 months after the third dose of the primary series mRNA COVID-19 vaccine, or at least 2 months after the additional primary dose following an initial Ad26.COV2.S dose).^16^

### Covariates

To determine the covariates included in this study, we considered factors found to be associated with vaccination uptake or completion in the literature^19,20^ as well as factors that could increase the risk for severe COVID-19 disease and thus influence vaccine uptake.^21,22^ Individual-level factors considered in this study included baseline sociodemographic characteristics (age, sex, and self-reported race and ethnicity recorded in the electronic health record, including Asian, Hispanic, non-Hispanic Black, non-Hispanic White, and other race and ethnicity [which included multiple races, Native American, and Pacific Islander], census tract level neighborhood, educational level, household income, and Medicaid status); history of SARS-CoV-2 infection; body mass index (BMI; calculated as weight in kilograms divided by height in meters squared: underweight, <18.5; normal, 18.5-24.9; overweight, 25.0-29.9; obese, ≥30.0); and Charlson Comorbidity Index (range with HIV excluded, 0-23, with higher values indicating greater comorbid disease burden) assessed during the 12 months prior to the index date. (Race and ethnicity were included because COVID-19 vaccine series completion rates may differ by race and ethnicity.) We further considered HIV disease factors (CD4 T cell count and HIV viral load), behavioral health diagnoses (mental health disorders, including mood disorders, bipolar disorders, and depression using *International Statistical Classification of Disease, Tenth Revision, *[*ICD-10*] codes F30-F39; and substance use disorders using *ICD-10* codes F10-F19), and health care utilization (number of outpatient or virtual visits, inpatient stay, and emergency department visits) in the 6 months prior to the index date; influenza vaccination in the 2 years before the index date, and VSD site.

To assess if the COVID-19 vaccine coverage among PWH is correlated with the level of COVID-19 vaccine uptake and disease burden in the general population in corresponding geographic areas, data on community-level COVID-19 vaccination coverage at the end of the study and the mean number of diagnosed COVID-19 cases per 100 000 persons in the county of residence during the study period were assessed using the CDC COVID-19 data tracker linked with geocoding data.^23^

### Statistical Analysis

We plotted the cumulative percentages of PWH who were partially vaccinated (ie, received 1 mRNA COVID-19 vaccine dose) and those who completed the primary vaccine series by calendar month through December 31, 2021. Similarly, among PWH who completed the primary series by August 12, 2021, the cumulative percentages of those who received the additional primary vaccine dose or a booster dose through April 30, 2022, were plotted. We conducted descriptive analyses of baseline characteristics, comparing the unvaccinated group with PWH who completed the primary series. Similar analyses were conducted among a subset of PWH who completed the primary series, comparing those having received an additional primary dose and those who did not. The Kruskal-Wallis test was conducted for continuous covariates, while the χ^2^ test or Fisher exact test was used for categorical covariates, as appropriate.

Separate multivariable analyses were conducted using a Poisson regression model with robust variance estimation^24^ to estimate the rate ratio (RR) and 95% CI and to identify factors associated with (1) the completion of the COVID-19 vaccine primary series (vs unvaccinated) in the overall cohort, and (2) the receipt of an additional primary dose (vs no additional primary dose) among persons who completed the primary series, respectively. Follow-up time was accounted for as an offset term in the models. All individual-level and community-level factors were included in the analysis. The *P* values were from 2-sided tests, and results were deemed statistically significant at a level of *P* < .05. All analyses were conducted with SAS, version 9.4 (SAS Institute Inc).

## Results

A total of 22 058 adult PWH who met the enrollment eligibility criteria were identified (mean [SD] age, 52.1 [13.3] years; 2471 [11.2%] female and 19 587 [88.8%] male; and 1201 [5.4%] Asian, 5908 [26.8%] Hispanic, 3687 [16.7%] non-Hispanic Black, 9386 [42.6%] non-Hispanic White, and 1876 [8.5%] categorized as other race and ethnicity) as of December 14, 2020. By December 31, 2021, 19 962 PWH (90.5%) completed the primary series, 1782 PWH (8.1%) received no COVID-19 vaccine dose, and 314 PWH (1.4%) received only 1 dose of an mRNA COVID-19 vaccine. The pattern of primary series coverage, by vaccine type, is presented in the Figure (panel A). Rapid uptake of the primary series was achieved within 6 months after the national COVID-19 vaccination program launched on December 14, 2020. A total of 18 374 eligible PWH (1770 [0.6%] female, 16 604 [90.4%] male) completed the primary series by August 12, 2021. Among them, 15 982 PWH (87.0%) received an additional primary dose, and 4318 PWH (23.5%) received a booster dose by April 30, 2022 (Figure, panel B). Rapid uptake of the additional primary dose was also observed after the FDA’s emergency use authorization for the additional primary dose, and uptake slowed after February 2022. Slow uptake of the booster dose began in November 2021 and seemed to increase in April 2022.

**Figure.**
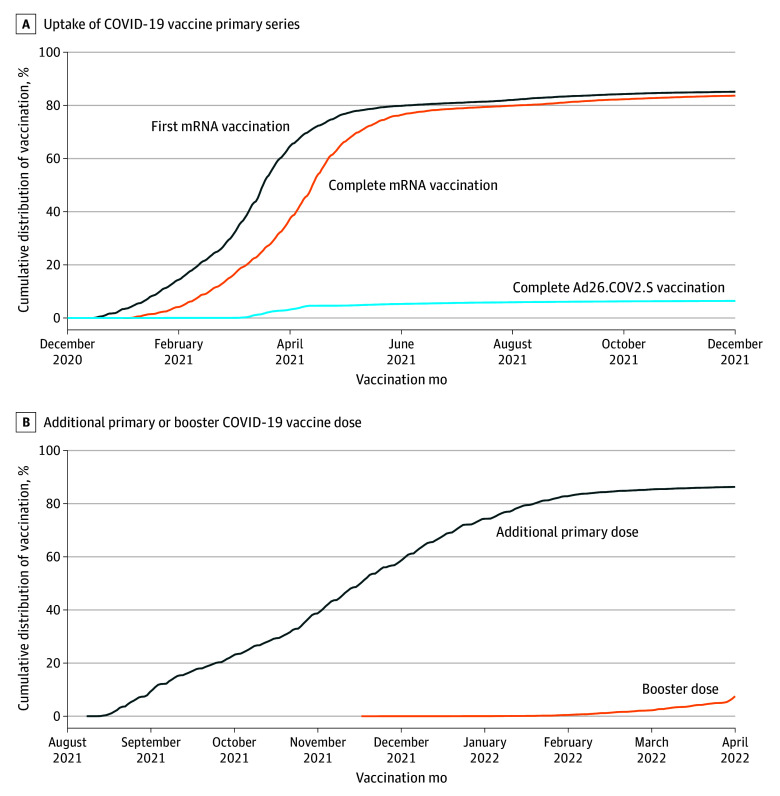
Uptake of COVID-19 Vaccination Primary Series and Additional Primary or Booster Dose Among Persons With HIV in the Vaccine Safety Datalink Uptake of COVID-19 primary series was assessed from December 14, 2020, through December 31, 2021. Uptake of an additional primary vaccination or booster dose was assessed from August 12, 2021, through April 30, 2022, among persons with HIV who completed the primary series. The index date was December 14, 2020, for the assessment of primary series completion, and August 12, 2021, for the assessment of uptake of additional primary or booster dose.

Table 1 summarizes baseline characteristics by the status of completion of the primary series. Compared with PWH with no vaccination, PWH who completed the primary series were older (mean [SD] age, 52.7 [13.0] years vs 45.4 [14.2] years; *P* < .001) and more likely to be male (89.7% vs 79.3%; *P* < .001) and non-Hispanic White (43.6% vs 32.9%; *P* < .001). In the multivariable analysis examining the association of multiple individual- and community-level factors simultaneously, the factor most strongly associated with completion of the primary series (compared with no vaccination) was having received an influenza vaccination in the last 2 years (RR, 1.17 [95% CI, 1.15-1.20]). Other factors associated with completion of the primary series included male sex (RR, 1.06 [95% CI, 1.05-1.08]), Asian race as compared with White race (RR, 1.05 [95% CI, 1.03-1.06]), higher neighborhood educational level (RR, 1.02 [95% CI, 1.01-1.03]), higher number of outpatient (in-person or virtual) care visits in the last 6 months (eg, RR, 1.07 [95% CI, 1.05-1.09] for ≥7 vs 0 visits), and residence in counties with a higher proportion of fully vaccinated adults (eg, RR, 1.06 [95% CI, 1.03-1.08] for fourth vs first quartiles) (eFigure 2A in Supplement 1). PWH aged 50 to 64 years were slightly more likely to complete the primary series compared with those aged 65 years or older (RR, 1.02 [95% CI, 1.01-1.03]), while PWH who were younger than 40 years were less likely to complete the primary series (RR, 0.87 [95% CI, 0.82-0.92] for ages 18-24 years; RR, 0.98 [95% CI, 0.96-0.99] for ages 25-39 years). PWH with compromised immune function (ie, CD4 count <500 cells/mm^3^) (RR, 0.98 [95% CI, 0.98-0.99] for <200 cells/mm^3^) and PWH with uncontrolled viremia (ie, HIV viral load ≥200 copies/mL) were less likely to complete the primary series (eg, RR, 0.90 [95% CI, 0.85-0.95] for viral load 200-10 000 copies/mL vs undetected or <200 copies/mL) (eFigure 2A in Supplement 1). Individuals who had Medicaid insurance were also less likely to complete the primary series (RR, 0.89 [95% CI, 0.87-0.90]).

**Table 1.** Baseline Characteristics of PWH by Completion of the COVID-19 Vaccine Primary Series or No COVID-19 Vaccination; Vaccine Safety Datalink, December 14, 2020, through December 31, 2021

Characteristic	PWH with no COVID-19 vaccination, No. (%) (n = 1782)	PWH with completed primary series, No. (%) (n = 19 962)^a^	Total, No. (%) (N = 21 744)	*P* value
Age, y^b^				
18-24	104 (5.8)	315 (1.6)	419 (1.9)	<.001
25-39	611 (34.3)	3518 (17.6)	4129 (19.0)	
40-49	349 (19.6)	3608 (18.1)	3957 (18.2)	
50-64	555 (31.1)	9153 (45.9)	9708 (44.6)	
≥65	163 (9.1)	3368 (16.9)	3531 (16.2)	
Sex				
Female	369 (20.7)	2053 (10.3)	2422 (11.1)	<.001
Male	1413 (79.3)	17 909 (89.7)	19 322 (88.9)	
Race and ethnicity				
Asian	34 (1.9)	1156 (5.8)	1190 (5.5)	<.001
Hispanic	500 (28.1)	5301 (26.6)	5801 (26.7)	
Non-Hispanic Black	455 (25.5)	3160 (15.8)	3615 (16.6)	
Non-Hispanic White	587 (32.9)	8706 (43.6)	9293 (42.7)	
Other or unknown^c^	206 (11.6)	1639 (8.2)	1845 (8.5)	
Medicaid insurance	507 (28.5)	1980 (9.9)	2487 (11.4)	<.001
Neighborhood-level median household income, $				
<40 000	221 (12.4)	1816 (9.1)	2037 (9.4)	<.001
40 000-79 999	1024 (57.5)	10 005 (50.1)	11 029 (50.7)	
≥80 000	487 (27.3)	7918 (39.7)	8405 (38.7)	
Missing	50 (2.8)	223 (1.1)	273 (1.3)	
Neighborhood-level education^d^				
<High school	538 (30.2)	4135 (20.7)	4673 (21.5)	<.001
≥High school	1193 (66.9)	15 598 (78.1)	16 791 (77.2)	
Missing	51 (2.9)	229 (1.1)	280 (1.3)	
BMI				
<18.5	12 (0.7)	107 (0.5)	119 (0.5)	<.001
18.5 to <25	1151 (64.6)	11 834 (59.3)	12 985 (59.7)	
25 to <30	332 (18.6)	4675 (23.4)	5007 (23.0)	
≥30	287 (16.1)	3346 (16.8)	3633 (16.7)	
Charlson Comorbidity Index^e^				
0	1276 (71.6)	12 249 (61.4)	13 525 (62.2)	<.001
1 to 2	358 (20.1)	5106 (25.6)	5464 (25.1)	
≥3	148 (8.3)	2607 (13.1)	2755 (12.7)	
No. of inpatient stays^f^				
0	1686 (94.6)	18 789 (94.1)	20 475 (94.2)	.72
1	71 (4.0)	909 (4.6)	980 (4.5)	
2	18 (1.0)	184 (0.9)	202 (0.9)	
≥3	7 (0.4)	80 (0.4)	87 (0.4)	
No. of emergency department visits^f^				
0	1520 (85.3)	17 692 (88.6)	19 212 (88.4)	<.001
1	165 (9.3)	1637 (8.2)	1802 (8.2)	
2	52 (2.9)	379 (1.9)	431 (2.0)	
≥3	45 (2.5)	254 (1.3)	299 (1.4)	
No. of outpatient or virtual visits^f^				
0	416 (23.3)	2316 (11.6)	2732 (12.6)	<.001
1-3	678 (38.1)	8211 (41.1)	8889 (40.9)	
4-6	304 (17.1)	4123 (20.7)	4427 (20.4)	
≥7	384 (21.5)	5312 (26.6)	5696 (26.2)	
Receipt of influenza vaccination in last 2 y	999 (56.1)	17 396 (87.1)	18 395 (84.6)	<.001
Mental health disorders^g^	305 (17.1)	3955 (19.8)	4260 (19.6)	.01
Substance use disorders^g^	305 (17.1)	2452 (12.3)	2757 (12.7)	<.001
History of SARS-CoV-2 infection	305 (17.1)	934 (4.7)	2757 (12.7)	.10
CD4 count, cells/mm^3^^h^				
<200	99 (5.6)	594 (3.0)	693 (3.2)	<.001
200-499	468 (26.3)	4848 (24.2)	5316 (24.4)	
≥500	985 (55.3)	13 171 (66.0)	14 156 (65.1)	
Missing	230 (12.9)	1349 (6.8)	1579 (7.3)	
HIV viral load, copies/mL^h^				
Undetected or <200	1410 (79.1)	18 814 (94.2)	20 224 (93.1)	<.001
200-10 000	81 (4.5)	270 (1.4)	351 (1.6)	
>10 000	84 (4.7)	261 (1.3)	345 (1.6)	
Missing	207 (11.6)	617 (3.1)	824 (3.8)	

Abbreviations: BMI, body mass index (calculated as weight in kilograms divided by height in meters squared); PWH, person with HIV.

^a^ Completion of the COVID-19 vaccine primary series is defined as 2 doses of mRNA COVID-19 vaccines (BNT162b2 or mRNA-1273) or 1 dose of Ad26.COV2.S.

^b^ Age was calculated at the index date (December 14, 2020).

^c^ Other captured all other race and ethnicity categories including multiple races, Native American, and Pacific Islander.

^d^ Defined as fewer than 50% or at least 50% of adults residing in the neighborhood who had more than a high school education.

^e^ Assessed in the 12 months prior to the index date; Charlson Comorbidity Index was calculated after excluding HIV disease from the algorithm (range, 0 to 23, with higher values indicating higher comorbidity burden).

^f^ Number of health care visits was assessed in the 6 months prior to the index date.

^g^ History of mental health and substance use disorders documented in the 6 months prior to the index date.

^h^ The CD4 count and HIV viral load measured within 6 months prior and closest to the index date.

Table 2 summarizes characteristics of PWH who completed an additional primary dose compared with those who did not. Demographic factors associated with additional primary dose completion were similar to those associated with completion of the primary series. In the multivariable analysis, the strongest factor associated with the completion of the additional primary dose (compared with no additional primary dose) was having received an influenza vaccination in the last 2 years (RR, 1.61 [95% CI, 1.54-1.69]), followed by male sex (RR,1.29 [95% CI, 1.23-1.36]). Other factors associated with the completion of the additional primary dose included higher neighborhood educational level (RR, 1.16 [95% CI, 1.12-1.21] for at least high school), higher Charlson Comorbidity Index (RR, 1.09 [95% CI, 1.04-1.15] for a score of ≥3), greater number of outpatient or virtual visits in the last 6 months (eg, RR, 1.21 [95% CI, 1.14-1.28] for ≥7 visits) and residence in counties with a greater proportion of adults fully vaccinated (eg, RR, 1.21 [95% CI, 1.14-1.29] for fourth vs first quartiles) (eFigure 2B in Supplement 1). PWH of younger age (18-24 years vs ≥65 years: RR, 0.36 [95% CI, 0.30-0.42]), PWH of non-Hispanic Black race and ethnicity (RR, 0.86 [95% CI, 0.83-0.89]) as compared with non-Hispanic White race (RR, 0.81 [95% CI, 0.77-0.84]), PWH who had Medicaid insurance (RR, 0.82 [95% CI, 0.78-0.87]), PWH with a history of SARS-CoV-2 infection (RR, 0.90 [95% CI, 0.86-0.94]), PWH with a history of substance use disorder in the last 6 months (RR, 0.90 [95% CI, 0.86-0.94]), and PWH who had a very high HIV viral load (RR, 0.65 [95% CI, 0.53-0.78] for viral load >10 000 copies/mL as compared with viral load <200 copies/mL) were less likely to complete the additional primary dose.

**Table 2.** Baseline Characteristics of PWH by Completion of an Additional COVID-19 Vaccine Primary Dose or No Additional Primary Dose; Vaccine Safety Datalink, August 12, 2021, Through April 30, 2022

Characteristic	PWH with no additional primary dose, No. (%) (n = 2392)	PWD with additional primary dose, No. (%) (n = 15 982)	Total, No. (%) (N = 18 374)	*P* value
Age, y^a^				
18-24	78 (3.3)	118 (0.7)	196 (1.1)	<.001
25-39	788 (32.9)	2130 (13.3)	2918 (15.9)	
40-49	498 (20.8)	2667 (16.7)	3165 (17.2)	
50-64	829 (34.7)	7768 (48.6)	8597 (46.8)	
≥65	199 (8.3)	3299 (20.6)	3498 (19.0)	
Sex				
Female	341 (14.3)	1429 (8.9)	1770 (9.6)	<.001
Male	2051 (85.7)	14 553 (91.1)	16 604 (90.4)	
Race and ethnicity				
Asian	110 (4.6)	974 (6.1)	1084 (5.9)	<.001
Hispanic	805 (33.7)	4027 (25.2)	4832 (26.3)	
Non-Hispanic Black	483 (20.2)	2267 (14.2)	2750 (15.0)	
Non-Hispanic White	736 (30.8)	7484 (46.8)	8220 (44.7)	
Other or unknown^b^	258 (10.8)	1230 (7.7)	1488 (8.1)	
Medicaid insurance in 2021	375 (15.7)	1345 (8.4)	1720 (9.4)	<.001
Neighborhood-level median household income, $				
<40 000	300 (12.5)	1368 (8.6)	1668 (9.1)	<.001
40 000-79 999	1308 (54.7)	7845 (49.1)	9153 (49.8)	
≥80 000	762 (31.9)	6665 (41.7)	7427 (40.4)	
Missing	22 (0.9)	104 (0.7)	126 (0.7)	
Neighborhood-level education^c^				
<High school	695 (29.1)	2995 (18.7)	3690 (20.1)	<.001
≥High school	1674 (70.0)	12 878 (80.6)	14 552 (79.2)	
Missing	23 (0.9)	109 (0.7)	132 (0.7)	
BMI				
<18.5	20 (0.8)	92 (0.6)	112 (0.6)	.01
18.5 to <25	1367 (57.1)	8652 (54.1)	10 019 (54.5)	
25 to <30	567 (23.7)	4233 (26.5)	4800 (26.1)	
≥30	438 (18.3)	3005 (18.8)	3443 (18.7)	
Charlson Comorbidity Index^d^				
0	1722 (72.0)	9289 (58.1)	11 011 (59.9)	<.001
1-2	464 (19.4)	4318 (27.0)	4782 (26.0)	
≥3	206 (8.6)	2375 (14.9)	2581 (14.0)	
No. of inpatient stays^e^				
0	2276 (95.2)	15 009 (93.9)	17 285 (94.1)	.01
1	84 (3.5)	762 (4.8)	846 (4.6)	
2	17 (0.7)	154 (1.0)	171 (0.9)	
≥3	15 (0.6)	57 (0.4)	72 (0.4)	
No. of emergency department visits^e^				
0	2063 (86.2)	14 067 (88.0)	16 130 (87.8)	.03
1	227 (9.5)	1374 (8.6)	1601 (8.7)	
2	57 (2.4)	336 (2.1)	393 (2.1)	
≥3	45 (1.9)	205 (1.3)	250 (1.4)	
No. of outpatient and virtual visits^e^				
0	249 (10.4)	1336 (8.4)	1585 (8.6)	<.001
1-3	984 (41.1)	5779 (36.2)	6763 (36.8)	
4-6	568 (23.7)	3892 (24.4)	4460 (24.3)	
≥7	591 (24.7)	4975 (31.1)	5566 (30.3)	
Receipt of influenza vaccination in last 2 y	1775 (74.2)	14 404 (90.1)	16 179 (88.1)	<.001
Mental health disorders^f^	405 (16.9)	3247 (20.3)	3652 (19.9)	<.001
Substance use disorders^f^	345 (14.4)	1912 (12.0)	2257 (12.3)	<.001
History of SARS-CoV-2 infection	312 (13.0)	1388 (8.7)	1700 (9.3)	<.001
CD4 count^g^, cells/mm^3^				
<200	80 (3.3)	394 (2.5)	474 (2.6)	.02
200-499	576 (24.1)	4023 (25.2)	4599 (25.0)	
≥500	1625 (67.9)	10 711 (67.0)	12 336 (67.1)	
Missing	111 (4.6)	854 (5.3)	965 (5.3)	
HIV viral load, copies/mL^g^				
Undetected or <200	2199 (91.9)	15 436 (96.6)	17 635 (96.0)	<.001
200-10 000	47 (2.0)	162 (1.0)	209 (1.1)	
>10 000	58 (2.4)	104 (0.7)	162 (0.9)	
Missing	88 (3.7)	280 (1.8)	368 (2.0)	

Abbreviations: BMI, body mass index (calculated as weight in kilograms divided by height in meters squared); PWH, person with HIV).

^a^ Age was calculated at the index date, August 12, 2021.

^b^ Other captured all other race and ethnicity categories, including multiple races, Native American, and Pacific Islander.

^c^ Defined as fewer than 50% or at least 50% of adults residing in the neighborhood who had a high school diploma or higher education.

^d^ Assessed in the 12 months prior to the index date; Charlson Comorbidity Index (range, 0-23, with higher values indicating greater comorbid disease burden) was calculated after excluding HIV disease from the algorithm.

^e^ Number of health care visits was assessed in the 6 months prior to the index date.

^f^ History of mental health and substance use disorders documented in the last 6 months prior to the index date.

^g^ The CD4 T cell count and HIV viral load measured within 6 months prior and closest to the index date.

## Discussion

In this cohort study using data from 8 health care organizations, rapid uptake and 90.5% COVID-19 vaccine primary series coverage were achieved among adult PWH by December 31, 2021. The completion rate of the primary series among insured PWH in our study was higher than that among the adult general population in the US (65%) reported by CDC as of October 13, 2021.^25^

The uptake of an additional primary dose was also high (87.0%) in our study. A large, matched cohort study conducted at Kaiser Permanente Southern California demonstrated that receipt of 3 doses of mRNA-1273 was associated with significantly higher relative vaccine effectiveness against SARS-CoV-2 infection and severe outcomes (compared with 2 doses) in immunocompromised populations,^26^ highlighting the importance of receiving 3 COVID-19 vaccine doses for immunocompromised populations. The rapid uptake of the additional primary dose observed in the present study suggests that significant morbidity and mortality may have been prevented in this vulnerable population.

Numerous factors, such as older age,^27,28^ male sex,^29,30^ and medical conditions, including overweight and obesity, diabetes, cardiovascular disease, cancer, chronic pulmonary disease, chronic kidney disease, and smoking history, have been reported as risk factors for severe COVID-19 disease in the general population without HIV.^7,31^ A recent cohort study reported higher odds of severe outcomes associated with low CD4 count, high viral load, and lack of antiretroviral therapy in PWH.^32^ In the present study, we observed higher COVID-19 coverage among older and male PWH. These findings suggest that the COVID-19 vaccination program may have prevented more severe COVID-19 disease in older PWH in the VSD sites. A higher baseline Charlson Comorbidity Index was associated with a higher completion of the additional primary dose. In addition, higher HIV viral load was associated with lower COVID-19 vaccine uptake even in this insured PWH population. This association may reflect the lack of engagement in routine HIV and preventive care in PWH with uncontrolled HIV viral load, suggesting more targeted vaccination outreach and routine care engagement efforts may improve COVID-19 vaccination coverage in this subset of PWH through more frequent interactions with the health system. Such outreach is of clinical and public health importance since among PWH, those without viral suppression are at increased risk of COVID-19 hospitalization.^6^ The associations with a history of influenza vaccination (in the prior 2 years) and number of outpatient or virtual visits (in the prior 6 months) suggest that utilization of routine health care, especially uptake of routine vaccination such as influenza vaccination, may be a good indicator for greater acceptance and uptake of a new vaccine. Completion of the primary series and receipt of the additional primary dose were also positively correlated with being resided in counties with a greater percentage of fully vaccinated adults. This finding suggests that promoting cultural acceptance of COVID-19 vaccines in the community in general and improving access to COVID-19 vaccines may improve vaccine uptake in high-risk populations, especially in the early months of the vaccination program. However, in this insured population, lower economic status remained an important factor contributing to inequity in COVID-19 vaccination. Our finding of the association between having Medicaid insurance and a lower likelihood of completion of the COVID-19 vaccine series is consistent with many studies reporting that individuals or households with low-income were less likely to receive COVID-19 vaccines,^33,34,35,36^ which may be partially due to living in areas with limited access to health facilities.

### Limitations

Several limitations need to be considered when interpreting the results of this study. First, vaccine coverage may be underestimated due to the limited follow-up time, especially for the booster dose. For assessing the coverage rate with the primary series and the additional primary dose, we allowed 1 year and 8 months, respectively, to complete the vaccination. We observed a plateau in uptake during the later months of the follow-up period for both the primary series and the additional primary dose. Second, for COVID-19 vaccines received outside of the health plan, there may be a delay or incomplete capture of vaccine doses. However, since efforts were made across VSD sites through routine integration of state immunization registry data,^37^ misclassification of vaccine status is anticipated to be low. Third, missing data for baseline HIV viral load and CD4 count may impact the interpretation of the results. The proportion of missing was higher in those who did not complete the vaccine primary series or additional primary dose, which may indicate missed routine HIV care visits and opportunities for vaccination. Finally, the high vaccination rates observed in our study may be due to the availability of more structured and accessible vaccination programs provided in these integrated health care systems. While our findings are likely generalizable to other commercially insured populations, COVID-19 vaccination coverage may be lower among those with less access to health care, including individuals who are uninsured.

## Conclusions

This cohort study found rapid uptake and high coverage with the COVID-19 vaccine primary series and additional primary dose among PWH with access to routine and preventive health care. Vaccination outreach efforts focusing on PWH who did not complete the series, particularly those with uncontrolled viremia, may help prevent morbidity and mortality from severe COVID-19 disease.

## Notes

Supplement 1. **eFigure 1.** Timeline for COVID-19 recommendations during the study period **eFigure 2.** Factors associated with (A) completion of the COVID-19 vaccine primary series and (B) additional primary dose among PWH in the Vaccine Safety Datalink, December 14, 2020, through April 30, 2021

Supplement 2.
Data Sharing Statement
