# Supplementary material for: COVID-19 Vaccination Coverage and Factors Associated With Vaccine Uptake Among People With HIV
Source: JAMA Netw Open. 2024 Jun 6;7(6):e2415220. doi: 10.1001/jamanetworkopen.2024.15220 (PMC11157350; doi:10.1001/jamanetworkopen.2024.15220)
Supplement: Supplement 1. — eFigure 1. Timeline for COVID-19 recommendations during the study period eFigure 2. Factors associated with (A) completion of the COVID-19 vaccine primary series and (B) additional primary dose among PWH in the Vaccine Safety Datalink, December 14, 2020, through April 30, 2021 [file jamanetwopen-e2415220-s001.pdf]

Supplementary Online Content

Hechter RC, Qian L, Liu ILA, Bree KK, Shan Y, Hensley PJ, et al. COVID-19 vaccination coverage and factors associated with vaccine uptake among people with HIV. *JAMA Netw Open*. 2024;7(6):e2415220. doi:10.1001/jamanetworkopen.2024.15220

eFigure 1. Timeline for COVID-19 recommendations during the study period

eFigure 2. Factors associated with (A) completion of the COVID-19 vaccine primary series and (B) additional primary dose among PWH in the Vaccine Safety Datalink, December 14, 2020, through April 30, 2021

This supplementary material has been provided by the authors to give readers additional information about their work.

© 2024 Hechter RC et al. *JAMA Network Open*.

eFigure 1. Timeline for COVID-19 recommendations during the study period

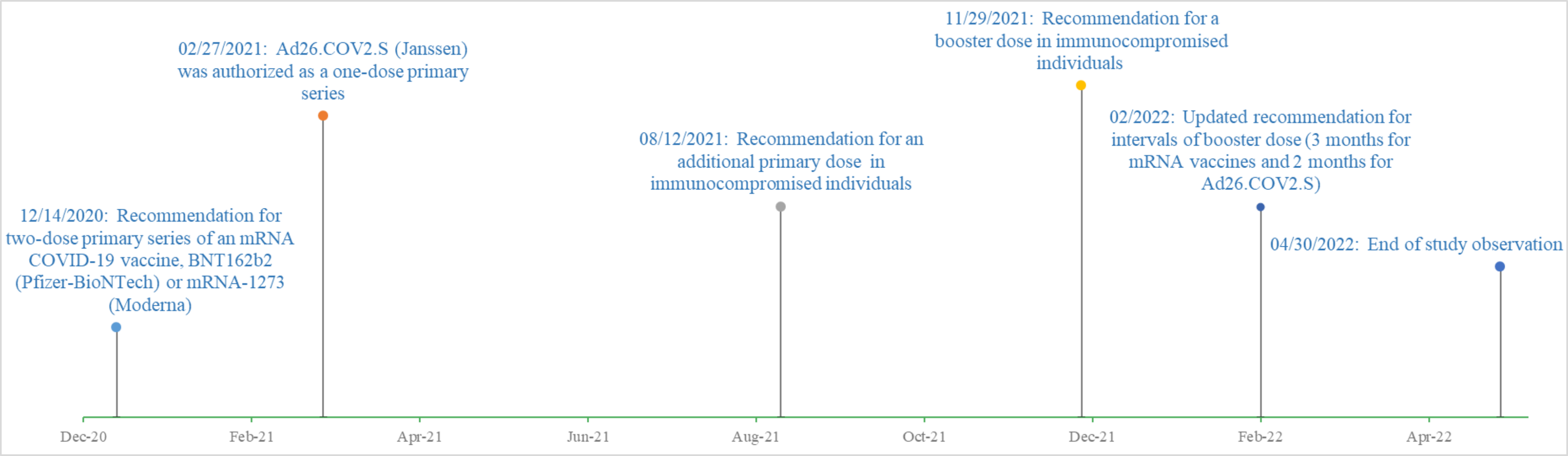

© 2024 Hechter RC et al. *JAMA Network Open*.

eFigure 2. Factors associated with (A) completion of the COVID-19 vaccine primary series and (B) additional primary dose among PWH in the Vaccine Safety Datalink, December 14, 2020, through April 30, 2021

© 2024 Hechter RC et al. *JAMA Network Open*.

Characteristics	a. Completion of Primary Series: Rate Ratio (95% CI)	b. Completion of Additional Primary Dose: Rate Ratio (95% CI)
Age[a], years		
18-24	0.87 (0.82-0.92)	0.36 (0.30-0.42)
25-39	0.98 (0.96-0.99)	0.52 (0.49-0.55)
40-49	1.00 (0.99-1.02)	0.69 (0.66-0.73)
50-64	1.02 (1.01-1.03)	0.80 (0.77-0.83)
≥65	Reference	Reference
Sex		
Female	Reference	Reference
Male	1.06 (1.05-1.08)	1.29 (1.23-1.36)
Race/ethnicity		
Asian	1.05 (1.03-1.06)	1.04 (0.98-1.10)
Hispanic	1.01 (1.00-1.02)	0.86 (0.83-0.89)
Non-Hispanic Black	0.99 (0.98-1.00)	0.81 (0.77-0.84)
Non-Hispanic White	Reference	Reference
Other[b]/Unknown	1.00 (0.99-1.02)	0.90 (0.85-0.95)
Medicaid Insurance		
No	Reference	Reference
Yes	0.89 (0.87-0.90)	0.82 (0.78-0.87)
Neighborhood-level median household income, $		
< 40,000	Reference	Reference
40,000-79,999	1.00 (0.99-1.02)	1.04 (0.99-1.10)
≥ 80,000	1.00 (0.99-1.02)	1.04 (0.98-1.10)
Missing	0.99 (0.76-1.30)	1.25 (0.58-2.72)
Neighborhood-level education[c]		
≤high school	Reference	Reference
>High school	1.02 (1.01-1.03)	1.16 (1.12-1.21)
Missing	0.96 (0.73-1.25)	0.92 (0.43-1.96)
BMI[d], km/m²		
<18.5	1.03 (0.97-1.09)	0.90 (0.75-1.07)
18.5-<25	Reference	Reference
25-<30	1.00 (0.99-1.01)	1.02 (0.99-1.05)
≥30	1.00 (0.99-1.01)	1.04 (1.00-1.08)
Charlson Comorbidity Index[d]		
0	Reference	Reference
1-2	1.00 (0.99-1.01)	1.06 (1.03-1.09)
≥3	1.00 (0.99-1.02)	1.09 (1.04-1.15)
No. of inpatient visits[e]		
0	Reference	Reference
1	1.01 (0.99-1.02)	1.06 (0.99-1.13)
2	1.00 (0.96-1.05)	1.06 (0.92-1.22)
≥3	1.04 (0.97-1.10)	0.90 (0.66-1.21)
No. of emergency department visits[e]		
0	Reference	Reference
1	1.00 (0.99-1.02)	0.94 (0.90-0.99)
2	0.98 (0.94-1.01)	0.89 (0.79-0.99)
≥3	0.96 (0.92-1.01)	0.78 (0.68-0.90)
No. of outpatient and virtual visits[e]		
0	Reference	Reference
1-3	1.05 (1.03-1.07)	1.03 (0.98-1.09)
4-6	1.06 (1.04-1.08)	1.10 (1.04-1.16)
≥7	1.07 (1.05-1.09)	1.21 (1.14-1.28)
Receipt of influenza vaccination in last 2 years		
No	Reference	Reference
Yes	1.17 (1.15-1.20)	1.61 (1.54-1.69)
Mental health disorders[f]		
No	Reference	Reference
Yes	1.01 (1.00-1.02)	1.04 (1.00-1.08)
Substance use disorders[f]		
No	Reference	Reference
Yes	0.99 (0.97-1.00)	0.90 (0.86-0.94)
History of SARS-CoV-2 infection		
No	Reference	Reference
Yes	1.00 (0.98-1.02)	0.90 (0.86-0.94)
CD4 count[g], cells/mm³		
<200	0.98 (0.98-0.99)	1.00 (0.97-1.04)
200-499	0.97 (0.94-0.99)	1.03 (0.92-1.14)
≥500	Reference	Reference
Missing	1.00 (0.99-1.02)	1.26 (1.17-1.35)
HIV viral load[g], copies/ml		
Undetected or <200	Reference	Reference
200-10,000	0.90 (0.85-0.95)	0.91 (0.78-1.06)
>10,000	0.92 (0.87-0.98)	0.65 (0.53-0.78)
Missing	0.89 (0.85-0.92)	0.72 (0.63-0.82)
Proportion of fully vaccinated adults in the county[h]		
First quartile (Q1)	Reference	Reference
Second quartile (Q2)	1.04 (1.02-1.05)	1.01 (0.96-1.06)
Third quartile (Q3)	1.01 (0.99-1.03)	1.07 (1.02-1.13)
Fourth quartile (Q4)	1.06 (1.03-1.08)	1.21 (1.14-1.29)
Number of COVID-19 cases/100K people in the county[i]		
First quartile (Q1)	Reference	Reference
Second quartile (Q2)	1.01 (0.99-1.03)	1.03 (0.99-1.08)
Third quartile (Q3)	1.03 (1.00-1.06)	1.14 (1.07-1.21)
Fourth quartile (Q4)	1.03 (1.00-1.06)	1.03 (0.96-1.09)

<-- Less Complete | More Complete -->

Axis a: 0.5, 1.0, 2.0; Axis b: 0.3, 0.5, 1.0, 2.0, 4.0

© 2024 Hechter RC et al. *JAMA Network Open*.

Completion of an additional primary dose was assessed among those who completed the primary series.
[a] Age was calculated at the index date, i.e., December 14, 2020 for the assessment of primary series completion and August 12, 2021 for the assessment of additional primary dose.
[b] Other captured all other race/ethnicity categories including multiple races, Native American, and Pacific Islander.
[c] Defined as < 50% or ≥ 50% of adults residing in the neighborhood who had high school diploma or higher education.
[d] Assessed in the 12 months prior to the index date; Charlson Comorbidity Index was calculated after excluding HIV disease from the algorithm.
[e] Number of health care visits were assessed in the 6 months prior to the index date.
[f] History of mental health and substance use disorders documented in the last 6 months prior to the index date.
[g] CD4 count and HIV viral load measured within 6 months prior and closest to the index date.
[h] Geocoding data were linked to the CDC COVID-19 data tracker at county level at the end of the study period.
[i] Estimated average number of diagnosed COVID-19 cases/100,000 people residing in the same county during the study period, using the CDC COVID-19 data tracker.

© 2024 Hechter RC et al. *JAMA Network Open*.
