# Supplementary material for: COVID-19 Vaccination Coverage and Factors Associated With Vaccine Uptake Among People With HIV
Source: JAMA Netw Open. 2024 Jun 6;7(6):e2415220. doi: 10.1001/jamanetworkopen.2024.15220 (PMC11157350; doi:10.1001/jamanetworkopen.2024.15220)
Supplement: Supplement 2. — Data Sharing Statement [file jamanetwopen-e2415220-s002.pdf]

Data Sharing Statement

Hechter. COVID-19 Vaccination Coverage and Factors Associated With Vaccine Uptake Among People With HIV. *JAMA Netw Open*. Published June 06, 2024.
doi:10.1001/jamanetworkopen.2024.15220

Data
Data available: No

Additional Information
Explanation for why data not available: The data that support the study conclusions are unavailable for public access. Guidelines on how to access VSD data through a sharing program administered by the National Center for Health Statistics Research Data Center (NCHSRDC) are provided here:
https://www.cdc.gov/vaccinesafety/ensuringsafety/monitoring/vsd/data-sharing-guidelines.html
and are subject to change.
